# Cost-effectiveness of Digital Tools for Behavior Change Interventions Among People With Chronic Diseases: Systematic Review

**DOI:** 10.2196/42396

**Published:** 2023-02-16

**Authors:** Tun Lin Kyaw, Nawi Ng, Margarita Theocharaki, Patrik Wennberg, Klas-Göran Sahlen

**Affiliations:** 1 Department of Epidemiology and Global Health Faculty of Medicine Umeå University Umeå Sweden; 2 School of Public Health and Community Medicine Institution of Medicine University of Gothenburg Göteborg Sweden; 3 Department of Public Health and Clinical Medicine Family Medicine Umeå University Umeå Sweden

**Keywords:** digital tools, chronic diseases, cost-effectiveness, lifestyle, behavior, systematic review, mobile phone

## Abstract

**Background:**

Chronic diseases, including cardiovascular diseases, diabetes, chronic obstructive pulmonary disease, and cerebrovascular diseases, contribute to the most significant disease burden worldwide, negatively impacting patients and their family members. People with chronic diseases have common modifiable behavioral risk factors, including smoking, alcohol overconsumption, and unhealthy diets. Digital-based interventions for promoting and sustaining behavioral changes have flourished in recent years, although evidence of the cost-effectiveness of such interventions remains inconclusive.

**Objective:**

In this study, we aimed to investigate the cost-effectiveness of digital health interventions for behavioral changes among people with chronic diseases.

**Methods:**

This systematic review evaluated published studies focused on the economic evaluation of digital tools for behavioral change among adults with chronic diseases. We followed the Population, Intervention, Comparator, and Outcomes framework to retrieve relevant publications from 4 databases: PubMed, CINAHL, Scopus, and Web of Science. We used the Joanna Briggs Institute’s criteria for economic evaluation and randomized controlled trials to assess the risk of bias in the studies. Two researchers independently screened, assessed the quality, and extracted data from the studies selected for the review.

**Results:**

In total, 20 studies published between 2003 and 2021 fulfilled our inclusion criteria. All the studies were conducted in high-income countries. These studies used telephones, SMS text messaging, mobile health apps, and websites as digital tools for behavior change communication. Most digital tools for interventions focused on diet and nutrition (17/20, 85%) and physical activity (16/20, 80%), and a few focused on smoking and tobacco control (8/20, 40%), alcohol reduction (6/20, 30%), and reduction of salt intake (3/20, 15%). Most studies (17/20, 85%) used the health care payer perspective for economic analysis, and only 15% (3/20) used the societal perspective. Only 45% (9/20) of studies conducted a full economic evaluation. Most studies (7/20, 35%) based on full economic evaluation and 30% (6/20) of studies based on partial economic evaluation found digital health interventions to be cost-effective and cost-saving. Most studies had short follow-ups and failed to include proper indicators for economic evaluation, such as quality-adjusted life-years, disability-adjusted life-years, lack of discounting, and sensitivity analysis.

**Conclusions:**

Digital health interventions for behavioral change among people with chronic diseases are cost-effective in high-income settings and can therefore be scaled up. Similar evidence from low- and middle-income countries based on properly designed studies for cost-effectiveness evaluation is urgently required. A full economic evaluation is needed to provide robust evidence for the cost-effectiveness of digital health interventions and their potential for scaling up in a wider population. Future studies should follow the National Institute for Health and Clinical Excellence recommendations to take a societal perspective, apply discounting, address parameter uncertainty, and apply a lifelong time horizon.

## Introduction

### Background

Chronic diseases are long-lasting conditions that do not improve or cure completely over time. Chronic diseases are the leading cause of death worldwide. According to the World Health Organization, ischemic heart disease, stroke, and chronic obstructive pulmonary disease (COPD) are the top 3 causes, whereas diabetes mellitus (DM) is the ninth leading cause of death globally [1]. In the Global Burden of Disease study (2016), disability-adjusted life-years (DALYs) because of ischemic heart disease, cerebrovascular disease, and lower respiratory infections accounted for 16.1% of all DALYs [2]*.* Approximately 10% of the adult population (≥40 years) had COPD [3]. In recent decades, the disease burden has shifted sharply toward noncommunicable diseases (NCDs) and injuries [4]. Between 1999 and 2019, ischemic heart disease, diabetes, stroke, chronic kidney disease, lung cancer, and age-related hearing loss showed the most substantial absolute increase in the number of DALYs, giving rise to the largest burden of disease in older age groups. Although there are several chronic diseases, this study focused on 4 major NCDs: cardiovascular diseases (CVDs), cerebrovascular diseases, COPD, and DM.

These chronic diseases share several risk factors, including tobacco use, unhealthy diet, physical inactivity, and excessive alcohol consumption [5]. The World Health Organization also highlighted that high systolic blood pressure (BP), tobacco use, dietary risks (eg, low intake of fruits and vegetables and high salt intake), air pollution, high fasting plasma glucose, high BMI, and high low-density lipoprotein cholesterol are the major risk factors responsible for millions of deaths worldwide [6]. Over the past decades, global exposure to several highly preventable risks has risen by >0.5% annually (obesity, high blood sugar, alcohol use, and drug use); these factors contribute not only to the growing burden of NCDs but also to the risk factors for a growing number of fatalities and highlight the necessity for investments in public health [7].

In addition to having direct consequences for persons with chronic diseases, chronic physical illnesses may also distort the lives of their families. A study in the Netherlands showed that chronic diseases negatively impact their partners in good health in 4 main areas: personal life, social relations, finance, and intrinsic rewards [8].

Today, smartphone use and internet access have increased significantly, providing the potential to improve health through the use of information technology. The term digital health intervention refers to interventions delivered using digital technologies such as smartphones, websites, and SMS text messages to provide effective, cost-effective, safe, and scalable interventions to enhance health and health care and promote healthy behaviors [9]. Developing complex health service interventions involves the use of behavior change techniques (BCTs). A BCT is “an observable, replicable, and irreducible component of an intervention designed to alter or redirect causal processes that regulate behavior, that is, a technique is proposed to be an active ingredient” [10]. In the National Institute for Health and Clinical Excellence (NICE) guidelines, interventions for changing unhealthy individual behaviors, such as unhealthy diet, physical inactivity, alcohol overconsumption, unsafe sexual practices, and smoking, are recommended to use evidence-based BCTs strategies such as goal-setting, feedback, and social support [11]. A previous systematic review and meta-analysis concluded that digital health interventions using smartphones, PCs, and wearable devices combined with technologies such as software, mobile apps, and the internet improve healthy behavioral factors such as physical activity (PA), diet, and medication compliance [12].

### Knowledge Gap

Despite the well-established evidence of behavioral lifestyle interventions on chronic disease–related morbidity and mortality, particularly when implemented at a population level or in high-risk groups [13], evidence on the cost-effectiveness of digital-based health interventions for NCD prevention and control is inconclusive. Available studies on economic analyses of digital health interventions have shown conflicting evidence and inconsistent findings. One systematic review published in 2002 argued that telemedicine is not a cost-effective method of delivering health care [14], whereas the systematic review by Rojas and Gagnon in 2008 confirmed that telemedicine is cost-effective in general, as it reduces hospital use and improves patient compliance, satisfaction, and quality of life [15]. To the best of our knowledge, there is no cost-effectiveness study combining digital tools and behavioral changes for chronic diseases. This study aimed to determine whether digital tools are cost-effective for lifestyle behavior interventions.

### Sustainable Behavior Change for Health Supported by Person-Tailored, Adaptive, Risk-Aware Digital Coaching in a Social Context Project

This study was part of the Sustainable Behavior Change for Health Supported by Person-Tailored, Adaptive, Risk-Aware Digital Coaching in a Social Context (STAR-C) project. It is an interdisciplinary research program aimed at developing and assessing a technical platform that can be used for behavior change interventions targeting CVD prevention through digital coaching. A team of researchers from complementary fields, such as public health, social science, computer science, cardiology, and health economics, designed and implemented this project [16]. The project will run in two phases from 2019 to 2024: (1) a formative intervention design and development phase and (2) an intervention evaluation phase. STAR-C will use gender and equity lenses in all phases of the program [17].

This study assessed the cost-effectiveness of digital health interventions for risk-reduction behavior and provided evidence-based recommendations regarding the health economic evaluation for the STAR-C project.

## Methods

### Overview

We conducted this review following the PRISMA (Preferred Reporting Items for Systematic Reviews and Meta-Analyses) guidelines. We used the Population, Intervention, Comparator, and Outcomes (PICO) framework to develop the review question to ensure that the relevant components of the question are well defined [18]. This review considered (1) studies that included adults with one or more of the 4 chronic diseases (CVDs, DM, COPD, and cerebrovascular diseases); (2) studies with economic evaluations using digital tools (telemedicine, mobile health [mHealth] apps, web-based, SMS text messaging, telephone consultations in combination with other digital support); and (3) studies that included behavior change interventions (quitting smoking, exercising optimally, taking a healthy diet, and reducing alcohol consumption). The comparators were no intervention, usual care, current practice, counselor-based counseling, or pharmacologic therapy. The following 4 major risk factors for chronic diseases were considered in this study: smoking or tobacco, overconsumption of alcohol, physical inactivity, and unhealthy diet (low intake of fruits and vegetables and excessive salt intake).

Studies were excluded if they were (1) systematic reviews or meta-analyses; (2) irrelevant publication types (editorials, letters, conference papers, commentary, case reports, study protocols, pilot studies, descriptive studies, and ecologic studies); (3) wrong study design (animal and in vitro trials and guidelines); (4) not published in English; (5) no information on outcomes (eg, pure economic studies without clinical or behavioral outcomes) or intervention costs (eg, those with only gross economic benefits were estimated); and (6) interventions using mass media, in addition to any deviation from PICO criteria.

### Types of Health Economic Evaluation

This review considers both partial and full health economic evaluations. According to Drummond, full economic evaluation is defined as a comparative analysis of alternative courses of action in terms of both their costs (resource use) and consequences (effectiveness), such as cost-benefit analysis (CBA), cost-effectiveness analysis (CEA), and cost-utility analysis (CUA) [19]. Partial economic evaluations either focus solely on costs or resource use without considering costs related to outcomes or focus on both costs and outcomes without comparing alternative interventions such as cost comparison or cost analysis, cost consequence analysis, cost description, outcome description, and cost of illness study [20].

### Search Strategies

We developed search strategies based on the PICO framework to retrieve the relevant publications. Accordingly, we created 4 separate search blocks, each based on one of the 4 topics: cost-effectiveness, behavior change, digital health intervention, and chronic conditions under study. Controlled vocabulary, including Medical Subject Headings and keywords, was also used in the search to ensure that as many relevant articles as possible were identified using synonyms and truncations in every search block. We used a Boolean operator to expand, exclude, or join keywords, using “AND” and “OR.” We searched the following 4 main bibliographic databases: PubMed, CINAHL, Scopus, and Web of Science. In addition to the web-based search, we manually conducted an extensive literature search using references from retrieved articles or recent results of ongoing studies identified from the database searches. Interested readers can find the detailed search blocks and terms in Multimedia Appendix 1.

### Study Selection

Initially, retrieved articles from the 4 databases were imported into Endnote on the web, a citation manager, where we removed duplicates before exporting the search results to Rayyan [21], a web-based platform to facilitate collaborative systematic review processes. First, we screened the titles and abstracts of all the search results, guided by our inclusion and exclusion criteria. If a paper was rejected, we recorded the reasons for exclusion. We downloaded all included articles for full-text reviews after the first screening. The full-text papers were again reviewed against the eligibility criteria (Multimedia Appendix 2). Two independent reviewers thoroughly scanned the titles, abstracts, and full texts. Reviewers then compared their independent decisions for inclusion, and disagreements during the review processes were resolved by discussion between the reviewers. The “blind on” option on Rayyan made it impossible to see the decision of another reviewer on a particular abstract, which helped reduce the risk of selection bias during screening.

### Data Extraction

We extracted data from each selected paper using a data-extraction form. These data included author, setting (country and year), inclusion and exclusion criteria, intervention and follow-up length of the study, number of participants in the intervention and control groups, economic perspective, uncertainty consideration (discounting and sensitivity analysis), outcomes, results, and type of behavioral interventions (Multimedia Appendix 3).

### Quality Review (Risk of Bias)

We appraised the quality of all included papers using the Joanna Briggs Institute criteria for economic evaluation and randomized controlled trials (RCTs; Multimedia Appendices 4 and 5). The economic quality criteria considered were the type of economic study, appropriate valuation of economic and clinical outcomes, uncertainty consideration (discounting), appropriate conclusions, and conflicts of interest. For the RCT criteria, this study considered the similarity of both groups at baseline, the same outcome in both groups, and the appropriate analysis. In terms of economic study design, this study rated full economic evaluation (CEA, CUA, and CBA) as *high quality* and others as *low quality*. A study was rated high quality if it used actual costs rather than estimated costs. The economic outcome of the study should be feasible for full economic analysis (eg, cost per quality-adjusted life-years [QALY] or DALY, cost per life-year saved, cost per clinical outcome, etc) to produce good quality. If the study period was >1 year, discounting should be included. This study used the NICE scale from the lowest to highest risk of bias to provide a qualitative appraisal [22]. The review used 10 criteria (a combination of economic and RCT criteria) for quality appraisal. Studies with ≥3 unfavorable responses (eg, *no*) were considered a *high risk* of bias. In comparison, we considered studies with 2 unfavorable responses a *medium risk*, and studies with 1 or no unfavorable responses were considered a *low risk*.

### Cost-effectiveness Appraisal

We assessed the cost-effectiveness of each study based on the cost-effectiveness threshold (CET) determined per country. Because it was impossible to determine the cost-effectiveness for partial economic studies, this study used the term *cost saving* or *not cost saving*, as stated in the respective study. The cost-effectiveness appraisal in this review was based entirely on conclusions of the respective studies.

## Results

### Study Selection and Characteristics

**Figure 1:**
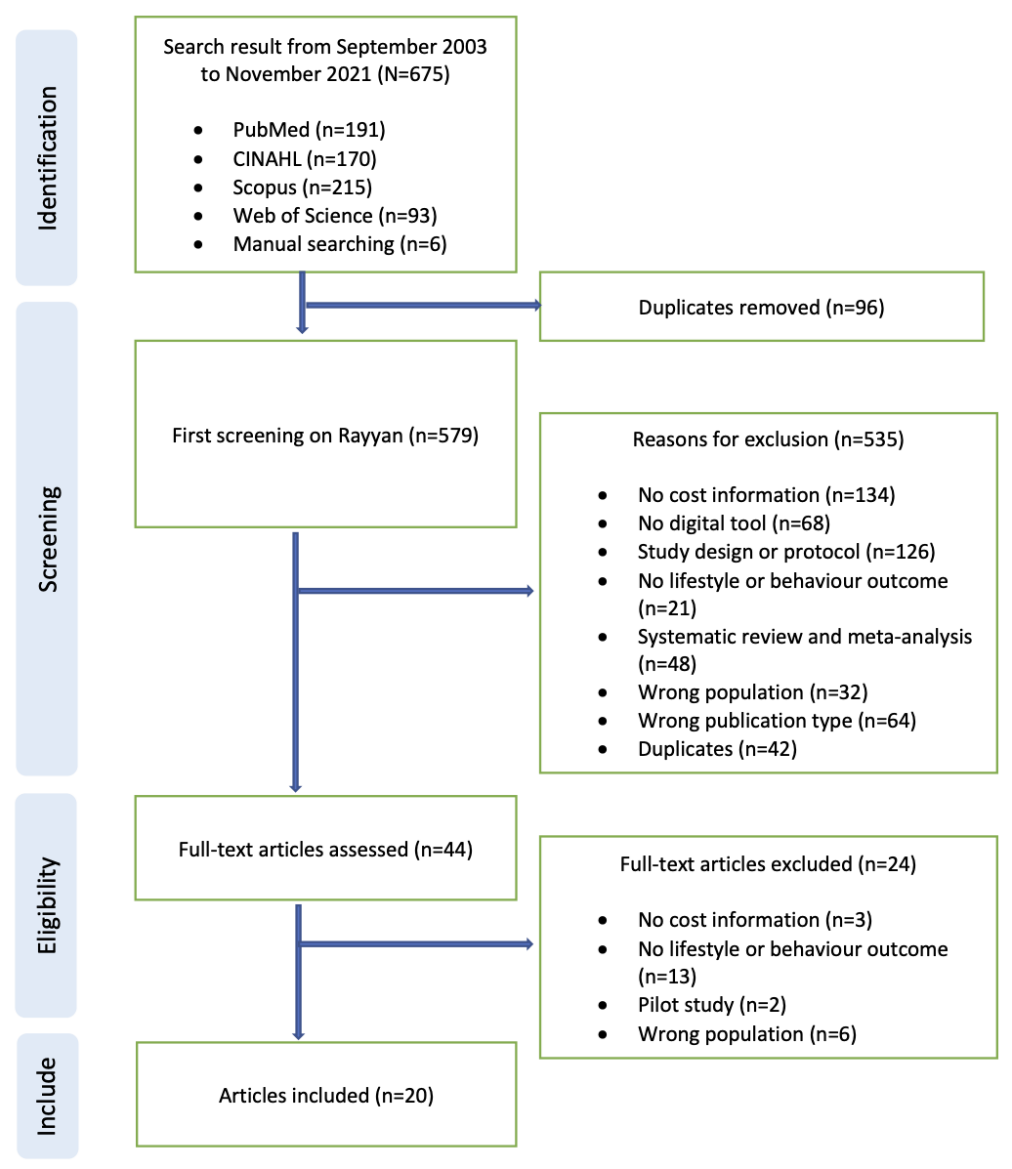
PRISMA (Preferred Reporting Item for Systematic Reviews and Meta-Analyses) flow diagram of studies showing reasons for exclusion.

A total of 675 papers appeared in the initial search results, of which 44 (6.5%) papers were eligible for full-text review, and 20 (3%) papers were included. In general, studies were excluded if they had no cost data, had no digital tools, had no lifestyle or behavior outcomes, had an inappropriate study design, or were study protocols (Figure 1).

#### Country of Origin

Most papers (12/20, 60%) in this review were from the United States [23-34]. The rest were from Australia (4/20, 20%) [35-38], New Zealand (1/20, 5%) [39], the United Kingdom (1/20, 5%) [40], Italy (1/20, 5%) [41], and 5% (1/20) of studies conducted in 3 countries (the Netherlands, Spain, and Taiwan) [42]. All the studies (20/20, 100%) were conducted in high-income countries. The period of publication of the studies ranged from 2003 to 2021; however, most studies (16/20, 80%) were published after 2010.

#### Disease Area and Patient Population

Most studies (12/20, 60% and 4/20, 20%) focused on CVD [24-26,29,32-35,38-40,42] (hypertension, ischemic heart disease, myocardial infarction [MI], and heart failure) and DM [28,30,31,37,41]. Overall, 15% (3/20) of other studies focused on CVD and DM [23,36,37], and only 5% (1/20) of studies focused on COPD [27]. No studies on cerebrovascular diseases were included, as none met the eligibility criteria. In most studies (18/20, 90%), the participants were those with one or more of the 4 chronic diseases. Furthermore, 10% (2/20) of studies [23,29] focused on people with a high risk of CVD and DM, measured by Framingham Risk Score [43], which included scoring on age, blood lipid profiles, smoking status, and hypertension (which is one of the subcategories of CVD).

Studies on DM included patients with type 1 diabetes (1/20, 5%), type 2 diabetes (2/20, 10%), and DM of nonspecific type. Generally, studies on individuals with severe diseases, complications, comorbidities, or who cannot exercise or have no mobile phone or internet access were excluded. The participants in these studies were aged 18 to 89 years, but 10% (2/20) of studies focused on the older adult (≥60 years) population [23,27].

#### Comparator

We included studies comparing digital health interventions with an alternative strategy representing the existing method of providing health services to the study population or on intervention. Most studies (15/20, 75%) compared key interventions with usual care, home health care, or existing practices. Some studies (3/20, 15%) used health education at the clinic, counselor-delivered counseling, or pharmacological therapies as comparators [26,29,30], and only 10% (2/20) of studies compared interventions with no intervention [23,35].

#### Study Design

Of the 20 studies, 9 (45%) performed full health economics analysis [25,29,30,35,36,38-40,42] using CEA and CUA methods, whereas the remaining 11 (55%) were partial economics studies. Furthermore, 60% (12/20) of studies used RCT design and incorporated economic evaluation. In full health economics studies (7/20, 35%), CUA, which used the QALY as the outcome measure, was the most common method. Only 35% (7/20) of studies [23,25,30,33,35,36,42] used modeling methods such as Markov modeling, event-based simulation, and decision trees. Other studies (13/20, 65%) were embedded in RCT studies.

#### Economic Perspective

An evaluation must specify and justify the perspective taken to measure behavior or lifestyle change programs and health resource use. A societal perspective is recommended by NICE [22], as the goal of public health is to improve the health and well-being of the whole population. Most studies (17/20, 85%) used health care payers as study perspectives, and only 15% (3/20) used a societal perspective [29,41,42].

#### Time Horizon

As this review focuses on chronic diseases, a longer time horizon is needed to measure the effects of costs and health outcomes. UK NICE guidelines prefers a lifetime horizon [22]. All studies (20/20, 100%) had a range of time horizons from 6 months to lifelong. In only 10% (2/20) of studies [35], the time horizon was a lifetime; in 10% (2/20) others [23,36], it was 10 years. Most studies (9/20, 45%) did not mention the time horizon, while for 30% (6/20) of studies, it was between 1 and 5 years; 5% (1/20) of studies used 6 months as the time horizon [38].

#### Direct Costs Included

Program-specific costs, a measure of program administration, program delivery, and program capital costs (eg, the technology needed for web-based interventions), are required. Health care costs, that is, the cost of all relevant health care services, such as general practitioner visits, specialist visits, hospitalizations, diagnostic tests and investigations, medications, and specialized equipment, must be calculated. The actual cost should be based on invoices, receipts, administrative records, and the hospital register rather than patient-estimated costs. All studies (20/20, 100%) in this review used both programs and direct medical costs in their calculations. Program costs differed significantly depending on the country, type, and year of intervention [24]. To make reading easier, all currencies other than US $ are always accompanied by the conversation to US $ (converted values in parentheses).

#### Indirect Costs Included

Studying costs from a societal perspective requires indirect costs, which include the patient’s or caregiver’s productivity loss owing to disease or travel time of the patient to health care services, as well as other home care costs. Of the 20 studies, only 3 (15%) studies [29,41,42] that used a societal perspective included the indirect costs.

#### Economic Outcomes

The incremental costs and outcomes of each health care program must be assessed in an economic evaluation. Accordingly, of the 20 studies, 7 (35%) studies using CUA methods presented incremental cost-effectiveness ratio (ICER) values based on the cost per QALY gained to assess the cost-effectiveness of the intervention. Furthermore, 10% (2/20) of other CEA studies showed ICER values using cost per life-year saved and cost per mm Hg reduction in BP. Although the remaining 55% (11/20) of studies did not provide cost-effectiveness information, it is still valuable to determine whether a treatment is justified based on its cost. Reduced use of health care resources is interpreted as evidence of improved outcomes in these studies, and it is usually presented as the average cost-savings per patient.

#### Sensitivity Analysis

Economic assessments should consider at least one sensitivity analysis to determine the robustness of the study results [44]. Nearly half of this review’s studies (9/20, 45%) performed sensitivity analyses, whereas the remaining studies (11/20, 55%) did not. Of the 9 studies with sensitivity analysis, 5 (56%) studies [25,35,36,39,40] performed probabilistic sensitivity analysis by the Monte Carlo simulation method.

#### Generalizability of the Result

Of the 20 studies, only 5 (25%) studies [28,35,37,40,42] discussed that their findings could be generalized to other populations, whereas the other 5 (25%) studies [25,26,29,34,36] did not. In the remaining studies (10/20, 50%), generalizability was not mentioned.

A description of the study characteristics, the economic perspective of the interventions, the results and the cost-effectiveness appraisal of selected studies can be found in Table 1.

**Table 1:** Study characteristics, economic perspective, results, and cost-effectiveness appraisal.

Study	Country and year	Study population	Follow-up^a^	Key intervention	Control	Perspective	Results	Cost-effectiveness
Bertuzzi et al [41]	Italy, 2017	Patients aged 5-50 years with type 1 DM^b^ and internet access	1 year	Teleconsultation, tele-education (nutrition, medication, and self-management; n=35)	Usual care (n=39)	Societal	No difference in HbA_1c_^c^ Reduced DM complications Saving of €80 per visit (US $89 per visit)	Inconclusive
Burn et al [35]	Australia, 2017	Patients with CHD^d^ or MI^e^ or bypass graft surgery	5 years	SMS Text message for behavior change over 24 weeks (n=5000)	No intervention	Health care	Reduced occurrence of MI and strokes ICER^f^: Aus $6123 per QALY^g^ (US $4,648 per QALY)	Cost-effective
Chen et al [23]	United States, 2016	Overweight or obese older adults (≥65 years) with risks for DM or CVD^h^ (by FRS^i^)	10 years	16 weeks of web-based education for behavior change (n=997)	No intervention	Health care	Saving of US $13,240 per capita at 10 years for prediabetes Saving of US $12,840 per capita at 10 years for pre-CVD	Cost-saving
Copeland et al [24]	United States, 2010	Patient ≥18 years with CHF^j^	1 year	Telephone coaching for behavior change (n=220)	Usual care (n=238)	Health care	No difference in clinical outcomes Higher total cost in the intervention group (US $6165) More regular exercise (OR^k^ 1.94, 95% CI 1.08-3.49)	Not cost-saving
Datta et al [25]	United States, 2010	Patients with hypertension and taking antihypertensive medication	2 years	Telephone coaching for behavior change (n=294)	Usual care (n=294)	Health care	No group difference in BP^l^ control ICER: US $42,457-US $87,300 per life-year saved	Cost-effective
Dunagan et al [26]	United States, 2005	Patient aged ≥21 years, at least one sign and symptom of heart failure	1 year	A phone call to improve self-management (diet and adherence to therapy) plus education about signs and symptoms of heart failure (n=76)	Education for heart failure (n=75)	Health care	Time to hospitalization (HR^m^ 0.67, 95% CI: 0.47-0.96) Hospital readmission (HR 0.67, 95% CI 0.46-0.99) Lowered hospital days and costs in the first 6 months only	Inconclusive
Finkelstein et al [27]	United States, 2006	Patients aged 60-96 years with CHF, chronic obstructive pulmonary disease, and chronic wound	2.5 years	Video group: HHC^n^ + 2 video consultations, Monitoring group: HHC + 2 video consultations +monitoring symptoms (n=54)	HHC (n=19)	Health care	No difference in mortality No difference in morbidity Lower cost than the control group	Cost-saving
Fischer et al [28]	United States, 2012	People aged >17 years with diabetes	20 months	Telephone coaching for medication management and healthy behavior (n=381)	Usual care (n=381)	Health care	LDL^o^ (AOR^p^ 1.72, 95% CI 1.28-2.32) Saving US $2433 per average patient cost No difference in the number of admissions	Cost-saving
Graves et al [36]	Australia, 2009	Adults with type 2 DM or hypertension	10 years	Telephone counseling over 1 year for physical activity and diet (n=228)	Existing practice (n=206)	Health care	ICER: Aus $29,375 per QALY gained (US $23,466 per QALY) CET^q^: 100% at a threshold of Aus $64,000 per QALY (US $51,126 per QALY)	Cost-effective
Hamar et al [37]	Australia, 2015	People aged 20-89 years with confirmed heart disease or DM; all under MGH^r^ program coverage	4 years	Telephone coaching and web-based tool for self-management and behavior change (n=4948)	Usual care (n=28,520)	Health care	Hospital admission rate (AOR 0.73, 95% CI 0.69-0.78) Readmission rate (AOR 0.55, 95% CI 0.48-0.63) Hospitalization days (ARR^s^ 0.83, 95% CI 0.77-0.90) Saving Aus $3549 per patient per year (US $2732 per patient per y)	Cost-saving
Keyserling et al [29]	United States, 2014	Adults aged 35-79 years with moderate to high risk for CVD (by FRS)	1 year	Web-based counseling for healthy behavior and medication adherence (n=193)	Counselor-delivered counseling (n=192)	Societal	No difference in FRS, ICER: US $2973 per QALY gained	Cost-effective
Maddison et al [39]	New Zealand, 2015	Patients with IHD aged ≥18 years and were able to perform the exercise	2 years	SMS text messaging and video messages via the website for exercise (n=85)	Usual care (n=86)	Health care	No difference in peaked O2 uptake More physical activity, More walking, ICER: US $28,768 per QALY gained	Cost-effective
McManus et al [40]	United Kingdom, 2021	People with poorly controlled hypertension	1 year	Web-based counseling for self-monitoring, titration of drugs, and healthy behaviors (n=305)	Usual care (n=317)	Health care	No group difference in BP ICER: £11 (US $13.27) per mm Hg reduction (95% CI £6-£29; [US $15] per mm Hg reduction)	Cost-effective
Nordyke et al [30]	United States, 2019	Patients aged 45-76 years with type 2 DM or hypertension	3 years	Digital therapeutic intervention using mobile phone app (n=2570)	Pharmacologic therapies (n=2575)	Health care	ICER: US $6468 per QALY for DM ICER: US $6620 per QALY for hypertension	Cost-effective
Nundy et al [31]	United States, 2014	people ≥18 years with DM	6 months	Text message for self-care and 2 weeks web education on diet, exercise and medication (n=74)	Usual care (n=274)	Health care	HbA1c level: group difference: −0.4% (*P*=.01) Cost-savings of US $437 per participant	Cost-saving
Piera-Jiménez et al [42]	The Netherlands, Spain, and Taiwan, 2020	Aged 18-75 years with hypertension or CHD or HF	5 years	SMS text messages and mobile apps for a healthy lifestyle over 6 months (n=120)	Usual care (n=118)	Societal	ICER: €124,489 per QALY (US $139,680 per QALY) in the Netherlands, €18,769 per QALY (US $21,059 per QALY) in Spain, €11,303 per QALY (US $12,682 per QALY) in Taiwan	Cost-effective for Spain, but not for the Netherlands and Taiwan
Southard et al [32]	United States, 2003	Patients with CHD or heart failure or both and access to the internet	6 months	Web-based education and email contact for exercise and diet over 6 months (n=53)	Usual care (n=51)	Health care	Fewer CVD events (15.7% reduction in intervention and 4.1% in the control group) Saving of US $1418 per patient	Cost-saving
Wang et al [33]	United States, 2012	Patients with poorly controlled hypertension, and taking drugs	18 months	Telephone intervention for 1. healthy behavior, 2. medication management, and 3. both (n=444)	Usual care (n=147)	Health care	No difference in BP control No difference in total costs	Not cost-saving
Maciejewski et al [34]	United States, 2014	Adults with hypertension medication and adults with poorly controlled hypertension	36 months	Telephone-delivered medication management, 2. software-assisted behavioral management, 3. combined over 18 months (n=444)	Usual care (n=147)	Health care	BP control: (17.1% patients; 95% CI 6.9-27.4) and US $3237 saving in behavioral arm, 20.2% patients (95% CI: 9.7-30.6) and US $977 saving in medication arm, and 20.4% patients (95% CI 10-30.8) and US $303 saving in the combined arm. No difference in cost-saving	Not cost-saving
Turkstra et al [38]	Australia, 2013	Patients aged 18-80 years with MI	1 years	Telephone coaching for self-monitoring, healthy behavior, and telemonitoring over 6 months (n=215)	Usual care (n=215)	Health care	No difference in HRQoL^t^ ICER: Aus $85,423 per QALY gained (US $82,072 per QALY)	Not cost-effective

^a^The follow-up time of trial.

^b^DM: diabetes mellitus.

^c^HbA_1c_: hemoglobin A_1c_.

^d^CHD: coronary heart disease.

^e^MI: myocardial infarction.

^f^ICER: incremental cost-effectiveness ratio.

^g^QALY: quality-adjusted life-years.

^h^CVD: cardiovascular disease.

^i^FRS: Framingham Risk Score.

^j^CHF: congestive heart failure.

^k^OR: odds ratio.

^l^BP: blood pressure.

^m^HR: hazard ratio.

^n^HHC: home health care.

^o^LDL: low-density lipoprotein.

^p^AOR: adjusted odds ratio.

^q^CET: cost-effectiveness threshold.

^r^MGH: My Health Guardian.

^s^ARR: adjusted risk ratio.

^t^HRQoL: health-related quality of life.

### Evidence for Cost-effectiveness

Of the studies (9/20, 45%) with full economic evaluation, 7 (78%) studies concluded that using digital tools for behavior modification was cost-effective when the comparators were no intervention, usual care, counselor-delivered counseling, or pharmacologic therapies [25,29,30,35,36,39,40]; 6 (86%) studies concluded their cost-effectiveness from the health care payer perspective and 1 (14%) from the societal perspective [29]. Of the studies (11/20, 55%) with partial economic evaluations, 55% (6/11) of studies were cost-saving; 18% (2/11) of studies were inconclusive [26,41]; and 27% (3/11) of studies were not cost-saving [24,33,34].

### Digital Tools for Intervention

The studies in this review used telephone, SMS text messaging, websites and software, mobile apps, and web-based video consultations as digital tools. The most cost-effective interventions (5/20, 25%) used telephone coaching, SMS text messaging, or health apps on mobile phones. Most studies (9/20, 45%) used telephones with other digital support as the tool for behavior change communication [24-26,28,33,34,36-38]. Typically, telephone interventions were provided by experienced nurses trained in motivational interviewing, but 22% (2/9) of these studies [36,38] used trained counselors and medical doctors.

Using a website to provide consultation or counseling for healthy behavior was the second most commonly used method in some studies (6/20, 30%) [23,29,32,34,40,41]. The studies involved a wide variety of health care professionals in web-based counseling. In addition, one study used email reminders to encourage exercise and incentives (key chains, athletic socks, book markers, and refrigerator magnets) to encourage active participation [32]; one study conducted in Italy used a website [45] for diabetes teleconsultation [41].

Another study used SMS text messaging for behavior change communication. Experts created automated messages that encouraged PA and a healthy diet for respective diseases and are typically sent out 3 to 5 times weekly [39]. In addition to behavior-related messages, they also reminded the patient about self-monitoring (eg, “time to check blood sugar”) [31].

The use of mobile apps, such as Moves, Vire, and Beddit, to encourage healthy behavior has been observed in 10% (2/20) of studies [30,42]. These apps were designed to integrate input from all monitoring devices, including pedometers that count steps, and the HORUS app collected pictures of the patients’ meals to provide dietary recommendations. These apps provided information to patients and create alerts for exercise [42]. Overall, 5% (1/20) of studies used video calls for internet-based visits and encouraged patients to exercise [27].

### Types of Risk Behaviors Aimed by Interventions

#### Smoking and Tobacco Control

Of the 8 (40%) studies on smoking cessation interventions, 5 (62%) were conducted in the United States [25,28,29,33,34], 2 (25%) in Australia [35,38], and 1 (12%) in 3 countries (the Netherlands, Spain, and Taiwan) [42]. In total, 50% (4/8) of studies [25,29,35,42] concluded that smoking cessation interventions were cost-effective. In cost-effective interventions, the studies used web-based counseling, SMS text messaging, and telephone counseling as tools for behavior change. The SMS text messaging intervention (TEXT ME) was cost-effective in an Australian study using Markov simulation, with an ICER of Aus $6123 per QALY (US $4648 per QALY) gained when compared with no intervention with the CET of Aus $64,000 per QALY (US $51,125 per QALY) [35]. A study in the United States was cost-effective at an ICER of US $2973 per QALY gained when web-based counseling was compared with counselor-based counseling, given that the CET was US $100,000 per QALY. Another study in the United States compared telephone coaching for behavior change with usual care using life-year saved as an outcome measure and concluded that the intervention was cost-effective at an ICER of US $42,457 per life-year saved for women and US $87,300 per life-year saved for men [25]. One study in 3 countries showed that the intervention was cost-effective only in Spain with the ICER of €18,769 per QALY (US $21,059 per QALY) and not in the Netherlands and Taiwan [42].

#### Alcohol Reduction

The cost-effectiveness of alcohol reduction interventions was evaluated in only 30% (6/20) of studies that focused on people with MI, DM, or poorly controlled hypertension as study participants. Only 10% (2/20) of studies [25,40] confirmed that telephone coaching or web-based counseling for healthy behavior was more cost-effective than usual care. A study in the United Kingdom reported that the intervention was cost-effective at an ICER of £11 per mm Hg reduction (US $15 per mm Hg) in BP when the willingness-to-pay threshold was £20 per mm Hg reduction (US $28 per mm Hg) [40].

#### Salt Intake

In total, 15% (3/20) of studies considered salt intake control in their interventions and aimed at people with poorly controlled hypertension [33,34,40], and only 33% (1/3) of those studies showed that it was cost-effective [40].

#### PA Assessment

Most studies (16/20, 80%) included PA (exercise, walking, dancing, gardening, yoga, etc) in their interventions. Of the 16 studies, we found 7 (44%) studies to be cost-effective when we compared web-based counseling, SMS text messaging, and telephone counseling with no intervention, usual care, or counselor-led counseling [25,29,30,35,36,39,40]. Among cost-effective interventions, they used the telephone [25,36], SMS text messaging [35,39], websites [29,40], and mobile apps [30] as digital tools to encourage PA.

In an Australian study, PA improvement was measured as moderate PA engagement for ≥5 days per week for at least 150 minutes each time. It was also estimated that the total cost of telephone counseling was Aus $570 (US $460) for the first year and Aus $410 (US $330) per year for the next 10 years, and it was cost-effective with an ICER of Aus $29,375 per QALY (US $23,466 per QALY) gained, given that the willingness-to-pay threshold is Aus $64,000 per QALY (US $51,125 per QALY) [36]. In total, 10% (2/20) of studies used SMS text messaging to encourage PA, such as “the more you eat, the more you need to exercise” [35,39]. One study in New Zealand reported that SMS text messaging encouraged more leisure time PA (110.2 minutes per week) and more walking (151.4 minutes per week) in the intervention group and was cost-effective at an ICER of US $28,768 per QALY gained [39].

Using mHealth apps for PA was cost-effective, as measured by the ICER of US $6468 per QALY gained and US $6620 per QALY gained for digital interventions targeting people with DM and people with hypertension, respectively, when compared with pharmacological therapy [30]. According to an Australian study published in 2013, telephone coaching for PA was not cost-effective for patients with MI [38].

Of the 35% (7/20) of partial economic studies for PA, 6 (86%) studies [23,27,28,31,37] showed cost-savings with a wide range of values depending on the type of digital tools and country.

#### Diet and Nutrition

Most studies (17/20, 85%) evaluated the cost-effectiveness of diet and nutritional interventions, and 35% (6/17) of these studies found them to be cost-effective. These interventions used a web-based coaching [30,40], telephone coaching [25,36], mobile apps [30], and SMS text messaging [35] as digital tools targeting CVD (MI, ischemic heart disease, and hypertension) and type 2 DM. It had the same ICER values as those for PA.

Studies on behavioral interventions using telephone coaching for healthy diet and nutrition reported intervention costs of US $112 per participant in the United States [25] and Aus $570 (US $460) per participant in Australia [36]. Overall, 5% (1/20) of studies used mobile apps and SMS text messaging to promote a healthy diet. The HORUS application was designed to collect pictures of different meals of the patient to provide dietary recommendations [42].

According to studies with partial economic evaluations, 25% (5/20) of interventions for a healthy diet were cost-saving, and the value of the savings was the same as that for PA [23,28,31,32,37]. Overall, 10% (2/20) of studies reported that it was not cost-saving because of higher use of health care services among patients with heart failure and hypertension in intervention groups compared with usual care [24,33].

### Risk of Bias Assessment

Table 2 presents the risk of bias across the selected studies. Four studies were deemed high risk [24,26,27,41], 6 medium risk [28,31-34,37], and 10 had a low risk of bias.

Nearly half of all studies (9/20, 45%) involved in this review had a potential conflict of interest because of stakeholder involvement in the analysis processes and unclear disclaimers [23,24,26,27,31-34,37]. Of these studies, 22% (2/9) had a serious risk of conflict of interest, as 1 author is the cofounder of mHealth Solutions company [31], and the other authors received consultation funds from pharmaceutical companies [34]. The remaining 55% (11/20) of studies were deemed to have no conflicts of interest. In total, 40% (8/20) of studies in this review showed cost-effective results without any conflicts of interest.

In this review, 20% (4/20) of studies [24,26,28,32] had unclear research questions regarding economic evaluation; 10% (2/20) of studies [27,41] had imprecise valuations, as they did not use actual costs in at least one of the cost categories.

**Table 2:** Quality appraisal (risk of bias assessment).

Study	Is the research question for economic evaluation?	Is the economic study design appropriate?	Are costs valued appropriately?	Are outcomes valued appropriately?	Was discounting applied?	Is the conclusion appropriate?	Is the conflict of interest disclosed?	Were groups similar at baseline?	Was the same outcome measured in both groups?	Is the analysis appropriate?	Risk of bias
Bertuzzi et al [41]	No	No	No	Yes	No	No	Yes	Yes	Yes	Yes	High
Burn et al [35]	Yes	Yes	Yes	Yes	Yes	Yes	Yes	N/A^a^	N/A	N/A	Low
Chen et al [23]	Yes	No	Yes	Yes	Yes	Yes	No	N/A	Yes	N/A	Low
Copeland et al [24]	No	No	Yes	Yes	No	Yes	No	Yes	Yes	Yes	High
Datta et al [25]	Yes	Yes	Yes	Yes	Yes	Yes	Yes	Yes	Yes	N/A	Low
Dunagan et al [26]	No	No	Yes	Yes	No	Yes	No	Yes	Yes	Yes	High
Finkelstein et al [27]	Yes	No	No	Yes	No	Yes	No	Yes	Yes	Yes	High
Fischer et al [28]	No	No	Yes	Yes	No	Yes	Yes	Yes	Yes	Yes	Medium
Graves et al [36]	Yes	Yes	Yes	Yes	Yes	Yes	Yes	Yes	Yes	Yes	Low
Hamar et al [37]	Yes	No	Yes	Yes	No	Yes	No	Yes	Yes	Yes	Medium
Keyserling et al [29]	Yes	Yes	Yes	Yes	No	Yes	Yes	Yes	Yes	Yes	Low
Maddison et al [39]	Yes	Yes	Yes	Yes	Yes	Yes	Yes	Yes	Yes	Yes	Low
McManus et al [40]	Yes	No	Yes	Yes	No	Yes	Yes	Yes	Yes	Yes	Low
Nordyke et al [30]	Yes	Yes	Yes	Yes	Yes	Yes	Yes	N/A	N/A	N/A	Low
Nundy et al [31]	Yes	No	Yes	Yes	No	Yes	No	Yes	Yes	Yes	Medium
Piera-Jiménez et al [42]	Yes	Yes	Yes	Yes	Yes	Yes	Yes	Yes	Yes	Yes	Low
Southard et al [32]	Yes	No	Yes	Yes	No	Yes	No	Yes	Yes	Yes	Medium
Wang et al [33]	Yes	No	Yes	Yes	No	Yes	No	Yes	Yes	Yes	Medium
Maciejewski et al [34]	Yes	No	Yes	Yes	No	Yes	No	Yes	Yes	Yes	Medium
Turkstra et al [38]	Yes	Yes	Yes	Yes	No	Yes	Yes	Yes	Yes	Yes	Low

^a^N/A: not applicable.

## Discussion

### Principal Findings

In general, digital health interventions for healthy behavior in people with chronic diseases are cost-effective, as all studies with cost-effective results have a low risk of bias. Previous studies have shown that digital interventions positively affect smoking, alcohol consumption, diet, and PA [46]. However, it is impossible to know how this effect will sustain for many years, as many studies had considerably short follow-up periods. Studies on digital interventions for reducing behavioral risks of CVD in nonclinical adult populations revealed that they were effective 6 months after the end of the intervention, and the interventions lost their effectiveness after 12 months, according to a scoping review [46]. It also concluded that the shorter duration of effect was due to a shorter follow-up period and intention-to-treat analysis.

In most cases, studies in this review used <2 years as a follow-up period, and only 10% (2/20) used lifelong time horizons for economic evaluation [25,35]. Except for 10% (2/20) of studies [27,37] that used >2 years as an intervention period, most studies used parameters from the short-term effects of interventions to construct cost-effectiveness estimates and extrapolation. The results could be misleading because some behaviors could relapse, such as smoking, PA, and eating habits, which could diminish the effectiveness of the intervention, and hence extrapolation could overestimate the effects. This problem is particularly prevalent in mathematical modeling that predicts the outcomes of interventions over a person’s lifetime because their parameters of economic impacts are based on a model of behavioral changes beyond the intervention period.

Most studies (6/20, 30%) with cost-effective or cost-saving results were published after 2010 [25,29,30,35,39,40]. With technology costs likely to have decreased in the recent years, digital health intervention costs could have been higher in the studies published before 2010 than in more recent ones; therefore, the cost-effectiveness of digital health interventions could be confounded by the year of publication. In Australia, 2 studies used telephone coaching as the intervention method. One study conducted in 2009 showed that the intervention cost was Aus $570 (US $460) per participant [36], whereas the other study showed that it was Aus $33 (US $25) per participant in 2017 [35].

Only half of studies (10/20, 50%) used specific clinical indicators, such as hemoglobin A_1c_ level, low-density lipoprotein cholesterol, and BP in mmHg, to measure clinical outcomes concerning the program’s effectiveness. Other studies (10/20, 50%) used more general indicators, such as hospital admission rates, readmission rates, length of hospital stay, mortality rates, morbidity rates, and health-related quality of life, and interpreted reductions in these indicators as well as reduced health care resource use as evidence of improved clinical outcomes. For instance, decreased hospital admission rates or reduced outpatient visits could be due to reasons other than the effectiveness of the program. In addition, except for 15% (3/20) of studies [29,36,39] that used specific behavioral indicators, improvement in behavior or lifestyle was usually measured by clinical outcomes in most studies. These findings could be problematic in interpreting the program’s effectiveness, as the improvement in clinical outcomes may be due to pharmacologic effects (antihypertensive medication, for instance) rather than adoption of healthy behaviors.

Although UK NICE guidelines strongly recommends a societal perspective for economic evaluations, it was implemented in only 15% (3/20) of studies [29,41,42], whereas the others (17/20, 85%) used health care payer perspectives. The results of an economic evaluation could be more cost-effective when conducted from a societal perspective, partly because the inclusion of homecare costs and productivity loss owing to illness significantly impact economic benefits. Furthermore, nonhealth outcomes, such as waiting time, time to diagnosis, and improved education and reassurance, should also be considered when assessing the cost-effectiveness of an intervention program.

Some behavior change interventions are embedded in telemonitoring, tele-education, or teleconsultation services that act as internet-based visits and enhance patient self-monitoring [27,38,40,47]. As a result, physical access to health care services would be reduced, but this does not necessarily mean reduced demand because of a healthy lifestyle. Therefore, researchers should be aware of this pitfall and use more specific indicators to measure the outcomes of healthy behaviors.

Although 25% (5/20) of studies [28,35,37,40,42] concluded that their results could be generalized to other settings, this is only possible for populations with high chronic disease prevalence because none of these interventions were aimed at the entire population. Because of the need for more information from low- and middle-income countries (LMICs), evidence-based recommendations are challenging to develop; however, digital health interventions also have potential. Although all studies were conducted in high-income settings, scaling up the digital health intervention in LMICs is feasible because of the high NCD burden and high population in these countries. Labrique et al [48] discussed that scaling up the digital health interventions in LMICs is possible under 5 conditions: involvement of end user inputs, engagement of all stakeholders in the developmental process, a good technical profile (simplicity, interoperability, and adaptability), well-established policy, and availability of appropriate infrastructure for digital health. The mHealth platforms will be more effective than other eHealth platforms because mobile phone use is on the rise, and smartphone adoption and use is ubiquitous not only in high-income countries but also in LMICs [49]. In addition, a systematic review found that mHealth can significantly modify health behavior as smartphones become more accessible to underserved and minority communities [50].

Owing to the demand for remote health services resulting from COVID-19, health care systems have implemented digital health and telemedicine solutions. Although telemedicine and digital solutions cannot replace all components of the health care experience, they offer certain advantages, such as the convenience of care, technology-assisted remote interaction, and increased accessibility to care, which can be crucial in managing chronic diseases [51]. Cost-effectiveness, accessibility to specialty services, and the ability to assist in alleviating physician shortages are key benefits of telemedicine, especially during COVID-19 [52]. Although health care professionals’ attitudes toward telemedicine were influenced by factors such as self-efficacy, performance expectations, and facilitating conditions, mHealth emerged as the most preferred mode of telemedicine, enabling health care systems to be integrated into telemedicine systems during pandemics in low-income countries [53].

### Recommendations for Further Research

On the basis of the findings of this review, the following recommendations are suggested:

1. The research question should include a cost-effectiveness assessment of the interventions for economic evaluation. Future studies should follow NICE recommendations to take a societal perspective, apply discounting, address parameter uncertainty, and apply a lifelong time horizon.
2. A full economic evaluation (CEA, CBA, and CUA) is needed to evaluate the cost-effectiveness of digital health interventions.
3. Researchers should use behavior-specific indicators such as walking time (minutes per week) for PA, urine nicotine testing for smoking, daily serving of fruits and vegetables, or plasma carotenoid index for diet, in addition to clinical indicators for the respective diseases.
4. Future research should be conducted on more diverse populations with chronic diseases to identify populations that can benefit the most from these interventions.
5. Assessment of the cost-effectiveness of digital interventions for behavioral change should include all stakeholders, including policy makers, implementers, and end users, to ensure that the final product is acceptable, scalable, feasible, and sustainable for wider implementation.

### Limitations

First, because most studies in this review sought to determine the effectiveness of digital health interventions based on clinical outcomes, economic evaluations were embedded in RCTs. Thus, most studies have many weaknesses in economic evaluations, such as not using QALY or DALY, no discounting, and no sensitivity analysis, which lead to uncertainty in decision making regarding cost-effectiveness. Moreover, this review contains no studies on LMICs, making it difficult to generalize the findings to broader regions because many LMICs have a poor infrastructure for digital health, such as an unstable internet connection. Finally, this review has limited conclusions owing to the heterogeneity of the interventions and diseases examined and the short follow-up periods. Furthermore, the heterogeneity of the results makes a meta-analysis difficult.

### Conclusions

Digital health interventions for behavioral change among people with chronic diseases are cost-effective in high-income settings and can therefore be scaled up. Similar evidence from LMICs based on properly designed studies for cost-effectiveness evaluation is urgently needed. A full economic evaluation is required to provide robust evidence of the cost-effectiveness of digital health interventions and their potential for scaling up in the broader population.

## Appendix

Multimedia Appendix 1 Search blocks.

Multimedia Appendix 2 Eligibility checklist.

Multimedia Appendix 3 Study characteristics.

Multimedia Appendix 4 Joanna Briggs Institute’s checklist for economic evaluations.

Multimedia Appendix 5 Joanna Briggs Institute’s checklist for randomized controlled trials.

## Abbreviations

BCT: behavior change technique
BP: blood pressure
CBA: cost-benefit analysis
CEA: cost-effectiveness analysis
CET: cost-effectiveness threshold
COPD: chronic obstructive pulmonary disease
CUA: cost-utility analysis
CVD: cardiovascular disease
DALY: disability-adjusted life-year
DM: diabetes mellitus
ICER: incremental cost-effectiveness ratio
LMICs: low- and middle-income countries
mHealth: mobile health
MI: myocardial infarction
NCD: noncommunicable disease
NICE: National Institute for Health and Clinical Excellence
PA: physical activity
PICO: Population, Intervention, Comparator, and Outcomes
PRISMA: Preferred Reporting Items for Systematic Reviews and Meta-Analyses
QALY: quality-adjusted life-years
RCT: randomized controlled trial
STAR-C: Sustainable Behavior Change for Health Supported by Person-Tailored, Adaptive, Risk-Aware Digital Coaching in a Social Context
