# Supplementary material for: Cost-effectiveness of Digital Tools for Behavior Change Interventions Among People With Chronic Diseases: Systematic Review
Source: Interact J Med Res. 2023 Feb 16;12:e42396. doi: 10.2196/42396 (PMC9982716; doi:10.2196/42396)
Supplement: Multimedia Appendix 1 [file ijmr_v12i1e42396_app1.docx]

**Research question:** Is using digital tools cost-effective for behaviour change in people with chronic diseases (CVD, Cerebrovascular, DM and COPD)?

**PICO framework**

**Population** People with one or more of four chronic diseases (CVD, Cerebrovascular, DM and COPD)

**Intervention** Behaviour change counselling using digital tools

**Comparator** Usual care, existing care, or no intervention

**Outcome** Behaviour change and cost-effectiveness

1. Database: Pubmed Date: November 2021

| **Element** | **Search** | **Search term** | **Results** |
| --- | --- | --- | --- |
| **P** | #1 | "cardiovascular diseases"[MeSH Terms] OR ("cardiovascular diseases"[MeSH Terms] OR ("cardiovascular"[All Fields] AND "diseases"[All Fields]) OR "cardiovascular diseases"[All Fields]) OR "heart diseases"[MeSH Terms] OR ("heart diseases"[MeSH Terms] OR ("heart"[All Fields] AND "diseases"[All Fields]) OR "heart diseases"[All Fields]) OR "cerebrovascular disorders"[MeSH Terms] OR ("cerebrovascular disorders"[MeSH Terms] OR ("cerebrovascular"[All Fields] AND "disorders"[All Fields]) OR "cerebrovascular disorders"[All Fields] OR ("cerebrovascular"[All Fields] AND "disease"[All Fields]) OR "cerebrovascular disease"[All Fields]) OR "stroke"[All Fields] OR "diabetes mellitus"[MeSH Terms] OR ("diabetes mellitus"[MeSH Terms] OR ("diabetes"[All Fields] AND "mellitus"[All Fields]) OR "diabetes mellitus"[All Fields]) OR "pulmonary disease, chronic obstructive"[MeSH Terms] OR ("pulmonary disease, chronic obstructive"[MeSH Terms] OR ("pulmonary"[All Fields] AND "disease"[All Fields] AND "chronic"[All Fields] AND "obstructive"[All Fields]) OR "chronic obstructive pulmonary disease"[All Fields] OR ("chronic"[All Fields] AND "obstructive"[All Fields] AND "pulmonary"[All Fields] AND "disease"[All Fields])) | 3,192,647 |
| **I** | #2 | "telemedicine"[MeSH Terms] OR ("telemedicine"[MeSH Terms] OR "telemedicine"[All Fields] OR "telemedicine s"[All Fields]) OR "telemedicine"[MeSH Terms] OR ("telemedicine"[MeSH Terms] OR "telemedicine"[All Fields] OR ("mobile"[All Fields] AND "health"[All Fields]) OR "mobile health"[All Fields]) OR "mobile applications"[MeSH Terms] OR ("mobile applications"[MeSH Terms] OR ("mobile"[All Fields] AND "applications"[All Fields]) OR "mobile applications"[All Fields] OR ("mobile"[All Fields] AND "application"[All Fields]) OR "mobile application"[All Fields]) OR ("telemedicine"[MeSH Terms] OR "telemedicine"[All Fields] OR ("mobile"[All Fields] AND "health"[All Fields]) OR "mobile health"[All Fields]) OR ("mhealth s"[All Fields] OR "telemedicine"[MeSH Terms] OR "telemedicine"[All Fields] OR "mhealth"[All Fields]) OR ("telemedicine"[MeSH Terms] OR "telemedicine"[All Fields] OR "ehealth"[All Fields]) | 102,965 |
| **C** |  |  |  |
| **O** | #3 | "behavior"[MeSH Terms] OR ("behavior"[MeSH Terms] OR "behavior"[All Fields] OR "behavioral"[All Fields] OR "behavioural"[All Fields] OR "behavior s"[All Fields] OR "behaviorally"[All Fields] OR "behaviour"[All Fields] OR "behaviourally"[All Fields] OR "behaviours"[All Fields] OR "behaviors"[All Fields] OR "pattern"[All Fields] OR "pattern s"[All Fields] OR "patternability"[All Fields] OR "patternable"[All Fields] OR "patterned"[All Fields] OR "patterning"[All Fields] OR "patternings"[All Fields] OR "patterns"[All Fields]) OR "health behavior"[MeSH Terms] OR ("health behaviour"[All Fields] OR "health behavior"[MeSH Terms] OR ("health"[All Fields] AND "behavior"[All Fields]) OR "health behavior"[All Fields]) OR "risk reduction behavior"[MeSH Terms] OR ("risk reduction behaviour"[All Fields] OR "risk reduction behavior"[MeSH Terms] OR ("risk"[All Fields] AND "reduction"[All Fields] AND "behavior"[All Fields]) OR "risk reduction behavior"[All Fields]) | 4,363,723 |
|  | #4 | "costs and cost analysis"[MeSH Terms] OR ("costs and cost analysis"[MeSH Terms] OR ("costs"[All Fields] AND "cost"[All Fields] AND "analysis"[All Fields]) OR "costs and cost analysis"[All Fields]) OR "cost benefit analysis"[MeSH Terms] OR ("cost benefit analysis"[MeSH Terms] OR ("cost benefit"[All Fields] AND "analysis"[All Fields]) OR "cost benefit analysis"[All Fields] OR ("cost"[All Fields] AND "benefit"[All Fields] AND "analysis"[All Fields]) OR "cost benefit analysis"[All Fields]) OR "cost benefit analysis"[MeSH Terms] OR ("cost benefit analysis"[MeSH Terms] OR ("cost benefit"[All Fields] AND "analysis"[All Fields]) OR "cost benefit analysis"[All Fields] OR ("cost"[All Fields] AND "utility"[All Fields] AND "analysis"[All Fields]) OR "cost utility analysis"[All Fields]) | 278,463 |
|  | #5 | #4 AND #2 | 3,605 |
|  | #6 | #5 AND #3 | 1,046 |
|  | #7 | #6 AND #1 | 191 |

1. Database: CINAHL Date: November 2021

| **Element** | **#** | **Search term** | **Results (Medline)** |
| --- | --- | --- | --- |
| **P** | #1 | ( cardiovascular disease or cvd or heart or cardiac or coronary heart disease ) OR ( cerebrovascular accident or cva or stroke ) OR diabetes mellitus OR ( chronic obstructive pulmonary disease or copd or chronic obstructive airway disease or chronic obstructive lung disease ) | 2,944,216 |
| **I** | #2 | ( telehealth or telemedicine or telemonitoring or telepractice or telenursing or telecare ) OR ( mhealth or mobile health or m-health or mobile app or mobile application ) OR ( ehealth or e-health or digital health ) | 142,415 |
| **C** |  |  |  |
| **O** | #3 | behaviour change OR health behavior change OR risk reduction behaviors | 158,611 |
|  | #4 | ( cost effectiveness or cost benefit or economics or cost management or economics ) OR cost effectiveness OR cost utility OR cost benefit | 932,529 |
|  | #5 | #4 AND #2 | 15,077 |
|  | #6 | #5 AND #3 | 709 |
|  | #7 | #6 AND #1 | 170 |

1. Database: Scopus Date: November 2021

| **Element** | **#** | **Search term** | **Results** |
| --- | --- | --- | --- |
| P | #1 | ( TITLE ( cardiovascular OR cvd OR heart AND disease ) OR TITLE ( cerebrovascular AND disease ) OR TITLE ( diabetes AND mellitus ) OR TITLE ( chronic AND obstructive AND pulmonary AND disease ) ) | 270,442 |
| I | #2 | ( TITLE-ABS-KEY ( telemedicine ) OR TITLE-ABS-KEY ( telehealth ) OR TITLE-ABS-KEY ( mobile AND health ) OR TITLE-ABS-KEY ( ehealth ) OR TITLE-ABS-KEY ( health ) OR TITLE-ABS-KEY ( mobile AND application ) ) | 5,423,756 |
| C |  |  |  |
| O | #3 | ( TITLE-ABS-KEY ( behaviour AND change ) OR TITLE-ABS-KEY ( health AND behaviour AND change ) OR TITLE-ABS-KEY ( risk AND reduction AND behavior ) ) | 790,543 |
|  | #4 | ( TITLE-ABS-KEY ( cost AND analysis ) OR TITLE-ABS-KEY ( cost AND effectiveness ) OR TITLE-ABS-KEY ( cost AND benefit ) OR TITLE-ABS-KEY ( cost AND utility ) ) | 1,431,484 |
|  | #5 | #4 AND #2 | 322,085 |
|  | #6 | #5 AND #3 | 8,407 |
|  | #7 | #6 AND #1 | 215 |

1. Database: Web of Science Date: November 2021

| **Element** | **#** | **Search term** | **Results** |
| --- | --- | --- | --- |
| **P** | #1 | (((TS=(cardiovasc* or cardiovascular disease )) OR TS=(cerebrovascular disease)) OR TS=(diabetes mellitus)) OR TS=(chronic obstructive pulmonary disease) | 1,170,694 |
| **I** | #2 | ((((TS=(Telemedic*)) OR TS=(Telehealth)) OR TS=(eHealth)) OR TS=(mHealth)) OR TS=(Mobile application) | 70,108 |
| **C** |  |  |  |
| **O** | #3 | ((TS=(behaviour change)) OR TS=(health behaviour change)) OR TS=(risk reduction behaviour) | 316,986 |
|  | #4 | (((TS=(cost*)) OR TS=(cost effectiveness)) OR TS=(cost benefit)) OR TS=(cost utility) | 789,559 |
|  | #5 | #4 AND #2 | 9,310 |
|  | #6 | #5 AND #3 | 446 |
|  | #7 | #6 AND #1 | 93 |

**Combination of four database**

| **Element** | **PubMed (Mesh + All)** | **CINAHL (Medline)** | **Scopus** | **Web of Science (Medline)** |
| --- | --- | --- | --- | --- |
| Population | 3,192,647 | 2,944,216 | 270,442 | 1,170,694 |
| Intervention | 102,965 | 142,415 | 5,423,756 | 70,108 |
| Comparator |  |  |  |  |
| Outcome | 4,363,723 | 158,611 | 790,543 | 316,986 |
|  | 278,463 | 932,529 | 1,431,484 | 789,559 |
|  | 3,605 | 15,077 | 322,085 | 9,310 |
|  | 1,046 | 709 | 8,407 | 446 |
|  | 191 | 170 | 215 | 93 |
| Manual search | 6 | | | |
| Total | **675** | | | |
