# Supplementary material for: Cost-effectiveness of Digital Tools for Behavior Change Interventions Among People With Chronic Diseases: Systematic Review
Source: Interact J Med Res. 2023 Feb 16;12:e42396. doi: 10.2196/42396 (PMC9982716; doi:10.2196/42396)
Supplement: Multimedia Appendix 2 [file ijmr_v12i1e42396_app2.docx]

1. **Study Design**

RCT □ Case-control □ Cohort □ Cross-sectional □

Full economic evaluation: CEA □ CUA □ CBA □ WTP □

Partial economic evaluation: ……………………………………………………………………………

**Participants**

| Criteria | Yes | No | Unclear |
| --- | --- | --- | --- |
| Adults (18yrs+) |  |  |  |
| Chronic disease of interest |  |  |  |

Notes ………………………………………………………………………………………………………………………………………………

1. Interventions

|  | Yes | No | Unclear |
| --- | --- | --- | --- |
| Using digital tools |  |  |  |

Notes ………………………………………………………………………………………………………………………………………………

1. Outcomes

|  | Yes | No | Unclear |
| --- | --- | --- | --- |
| Clinical outcome measure (including QoL) |  |  |  |
| Economic outcome measure |  |  |  |
| Intervention is lifestyle/behavioural |  |  |  |
| Inclusion of comparator |  |  |  |

Notes ………………………………………………………………………………………………………………………………………………

…………………………………………………………………………………………………………………………………………
