# Supplementary material for: Cost-effectiveness of Digital Tools for Behavior Change Interventions Among People With Chronic Diseases: Systematic Review
Source: Interact J Med Res. 2023 Feb 16;12:e42396. doi: 10.2196/42396 (PMC9982716; doi:10.2196/42396)
Supplement: Multimedia Appendix 3 [file ijmr_v12i1e42396_app3.docx]

| Study (author) | Country & Year | Inclusion criteria | Exclusion criteria | Intervention length | Follow up length | Participants in intervention | Participants in control | Total | Key intervention | Control | Perspective | Discounting | Sensitivity analysis | Outcomes | Results | Type of behaviour interv |
| --- | --- | --- | --- | --- | --- | --- | --- | --- | --- | --- | --- | --- | --- | --- | --- | --- |
|  |  |  |  |  |  |  |  |  |  |  |  |  |  |  |  |  |
|  |  |  |  |  |  |  |  |  |  |  |  |  |  |  |  |  |
|  |  |  |  |  |  |  |  |  |  |  |  |  |  |  |  |  |
|  |  |  |  |  |  |  |  |  |  |  |  |  |  |  |  |  |

Study characteristics

Note: type of behavioral intervention – smoking cessation, alcohol reduction, reduction in excessive salt intake, more physical activity, and healthy diet.
