# Supplementary material for: Cost-effectiveness of Digital Tools for Behavior Change Interventions Among People With Chronic Diseases: Systematic Review
Source: Interact J Med Res. 2023 Feb 16;12:e42396. doi: 10.2196/42396 (PMC9982716; doi:10.2196/42396)
Supplement: Multimedia Appendix 4 [file ijmr_v12i1e42396_app4.docx]

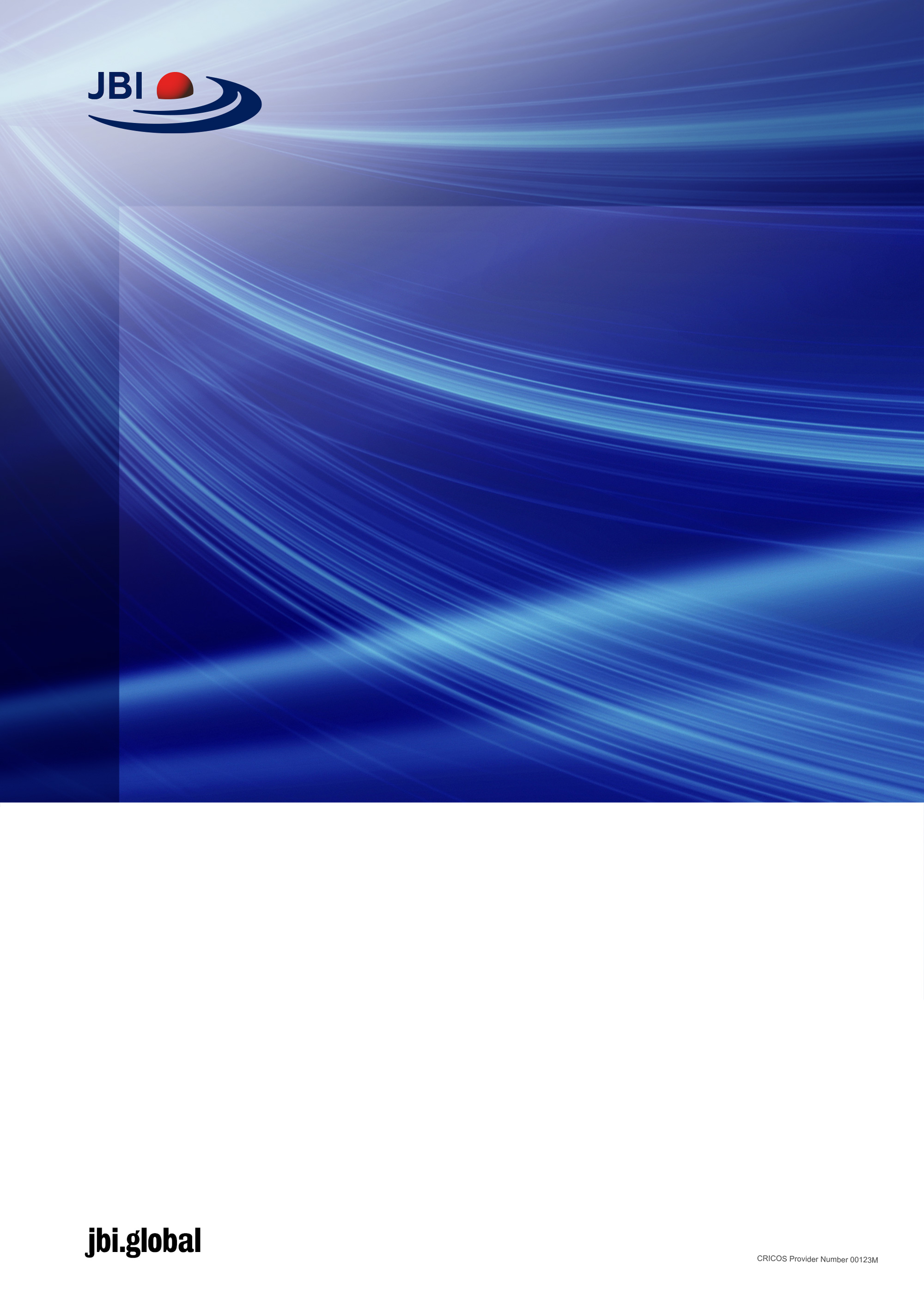


checklist for economic evaluations

Critical Appraisal tools for use in JBI Systematic Reviews

Introduction

JBI is an international research organisation based in the Faculty of Health and Medical Sciences at the University of Adelaide, South Australia. JBI develops and delivers unique evidence-based information, software, education and training designed to improve healthcare practice and health outcomes. With over 70 Collaborating Entities, servicing over 90 countries, JBI is a recognised global leader in evidence-based healthcare.

## JBI Systematic Reviews

The core of evidence synthesis is the systematic review of literature of a particular intervention, condition or issue. The systematic review is essentially an analysis of the available literature (that is, evidence) and a judgment of the effectiveness or otherwise of a practice, involving a series of complex steps. JBI takes a particular view on what counts as evidence and the methods utilised to synthesise those different types of evidence. In line with this broader view of evidence, JBI has developed theories, methodologies and rigorous processes for the critical appraisal and synthesis of these diverse forms of evidence in order to aid in clinical decision-making in healthcare. There now exists JBI guidance for conducting reviews of effectiveness research, qualitative research, prevalence/incidence, etiology/risk, economic evaluations, text/opinion, diagnostic test accuracy, mixed-methods, umbrella reviews and scoping reviews. Further information regarding JBI systematic reviews can be found in the [JBI Evidence Synthesis Manual](https://jbi-global-wiki.refined.site/space/MANUAL).

## JBI Critical Appraisal Tools

All systematic reviews incorporate a process of critique or appraisal of the research evidence. The purpose of this appraisal is to assess the methodological quality of a study and to determine the extent to which a study has addressed the possibility of bias in its design, conduct and analysis. All papers selected for inclusion in the systematic review (that is – those that meet the inclusion criteria described in the protocol) need to be subjected to rigorous appraisal by two critical appraisers. The results of this appraisal can then be used to inform synthesis and interpretation of the results of the study. JBI Critical appraisal tools have been developed by the JBI and collaborators and approved by the JBI Scientific Committee following extensive peer review. Although designed for use in systematic reviews, JBI critical appraisal tools can also be used when creating Critically Appraised Topics (CAT), in journal clubs and as an educational tool.

JBI Critical Appraisal Checklist for
economic evaluations

Reviewer ______________________________________ Date_______________________________

Author_______________________________________ Year_________ Record Number_________

|  | Yes | No | Unclear | Not applicable |
| --- | --- | --- | --- | --- |
| 1. Is there a well-defined question? | □ | □ | □ | □ |
| 1. Is there comprehensive description of alternatives? | □ | □ | □ | □ |
| 1. Are all important and relevant costs and outcomes for each alternative identified? | □ | □ | □ | □ |
| 1. Has clinical effectiveness been established? | □ | □ | □ | □ |
| 1. Are costs and outcomes measured accurately? | □ | □ | □ | □ |
| 1. Are costs and outcomes valued credibly? | □ | □ | □ | □ |
| 1. Are costs and outcomes adjusted for differential timing? | □ | □ | □ | □ |
| 1. Is there an incremental analysis of costs and consequences? | □ | □ | □ | □ |
| 1. Were sensitivity analyses conducted to investigate uncertainty in estimates of cost or consequences? | □ | □ | □ | □ |
| 1. Do study results include all issues of concern to users? | □ | □ | □ | □ |
| 1. Are the results generalizable to the setting of interest in the review? | □ | □ | □ | □ |

Overall appraisal: Include □ Exclude □ Seek further info □

Comments (Including reason for exclusion)

________________________________________________________________________________________________________________________________________________________________________________________________________________________________________________________________________________________________

jbi critical appraisal checklist for
economic evaluations

*How to cite: Gomersall JS, Jadotte YT, Xue Y, Lockwood S, Riddle D, Preda A. Conducting systematic reviews of economic evaluations. Int J Evid Based Healthc. 2015;13(3):170–178.*

This tool is informed by the work of Drummond et al, Methods for the economic evaluation of health care programmes. 2nd Edition. Oxford: Oxford Medical Publications, 1997.

## Is there a well-defined question/objective?

Consider the following before marking the study as compliant with this quality criterion:

- Is the objective/question of the study clearly stated?
- Does the statement reflect the perspective (e.g. patient or community or societal or health provider) used in measurement of costs or/and cost effectiveness?
- Was the study placed in a particular decision making context?

## Is there a comprehensive description of alternatives?

To be marked as compliant with this criterion the authors of the study should offer a clear description of the intervention or interventions considered in the economic evaluation and the comparator or comparators. Compliance does not require that a broad range of interventions and comparators was considered. What is important here is clear description of the nature of the intervention and comparator whose cost/effeteness was measured.

## Are all important and relevant costs and outcomes for each alternative identified?

This quality criterion assesses the comprehensiveness and relevant of the cost and cost effectiveness outcomes measured in the economic evaluation. When deciding whether all important costs and outcomes have been identified/measured in the study reflect on whether the outcomes are sufficient in light of the objectives of the study. It is appropriate for a study that has the objective of measuring a narrow range of costs and benefits to identify and measure a limited range. However, the limits of the narrow approach should be drawn out in the study. It is not appropriate for a study which implies in its objective statement that it measures a broad range of costs for a broad range out outcomes to include only a very limited range of relevant costs and outcomes.

## Has clinical effectiveness been established?

To assess compliance with this quality criterion requires considering whether the study has reported the evidence used to derive the effectiveness estimate and the level of this evidence. If it is not clear how the effectiveness estimate was derived, the study cannot be marked as compliant. To achieve compliance for this criterion the effectiveness estimate in the evaluation does not need to be derived from the same study as the resource use/cost estimate. What is important is the there is a solid evidence base under-pinning the assumptions about the direction and magnitude of the effectiveness measure(s) used in the evaluation.

## Are costs and outcomes measured accurately?

This quality criterion assesses whether the study has used appropriate/best practice measurement method to measure costs and effectiveness. To decide whether a study should be marked as compliant consider whether the methods section of the paper offers a detail description of the measures used for costs and outcomes and how it justifies them. In addition, consider whether the authors/study implementers discussed any limitations associated with the measures used and concerns about the accuracy of measurement. In economic evaluations it is often difficult to measure costs and outcomes accurately, and hence in many cases this quality criterion will be difficult to achieve.

## Are costs and outcomes valued credibly?

This quality criterion assesses whether appropriate prices were used to value costs and the validity of the valuation of benefits. It requires considering the method description and judging where there is a sufficient explanation about how costs and outcomes were valued and whether the justification for it is persuasive.

## Are costs and outcomes adjusted for differential timing?

To be marked compliant for this question the study should have identified and justified the discount rate used. The time frame over which the study was conducted should also have been identified and justified.

## Is there any incremental analysis of costs and consequences?

To achieve compliance the paper should report a measure that shows the change in costs and benefits for the intervention and comparator for a marginal shift in resources from the comparator to the intervention.

## Were sensitivity analysis conducted to investigate uncertainty in estimates of costs or outcomes?

Sensitivity analysis is critical for establishing the validity of any economic evaluations results. To be compliant a study must present sensitivity testing results that describe how the study findings vary with changes in key variables (for example relative prices, and intervention estimates? conducted to check the robustness of findings.

## Do study results include all issues of concern to users?

This question reflects on the comprehensiveness of coverage in the reporting of results. In deciding whether to mark the study as compliance consider whether the range of measures presented provider answers to all the questions users/decision makers would want to know when taking a decision about whether to implement the program examined (or cutting it)?

## Are the results generalizable to the setting of interest in the review?

To be marked as compliant for this last quality criterion the paper should: (i) have described the study setting adequately; (ii) discuss the issue of transferability of findings and how the results are generalizable to other settings with similar characteristics
